# Usefulness of C-reactive protein testing in acute cough/respiratory tract infection: an open cluster-randomized clinical trial with C-reactive protein testing in the intervention group

**DOI:** 10.1186/1471-2296-15-80

**Published:** 2014-05-02

**Authors:** Elena Andreeva, Hasse Melbye

**Affiliations:** 1 Department of Family Medicine, Northern State Medical University, Arkhangelsk, the Russian Federation; 2 Department of Community Medicine, Faculty of Health Sciences, The Arctic University of Norway, Tromsø, Norway

**Keywords:** Antibiotics, Respiratory tract infection, C-reactive protein, Chest radiography

## Abstract

**Background:**

Point of care testing for C-reactive protein (CRP) has shown promise as a measure to reduce unnecessary antibiotic prescribing in respiratory tract infections (RTI), but its use in primary care is still controversial. We aimed to evaluate the effect of CRP testing on the prescription of antibiotics, referral for radiography, and the outcome of patients in general practice with acute cough/RTI.

**Methods:**

An open-cluster randomized clinical trial was conducted, with CRP testing performed in the intervention group. Antibiotic prescribing and referral for radiography were the main outcome measures.

**Results:**

A total of 179 patients were included: 101 in the intervention group and 78 in the control group. The two groups were similar in clinical characteristics. In the intervention group, the antibiotic prescribing rate was 37.6%, which was significantly lower than that in the control group (58.9%) (P = 0.006). Referral for chest X-ray was also significantly lower in the intervention group (55.4%) than in the control group (75.6%) (P = 0.004). The recovery rate, as recorded by the GPs, was 92.9% and 93.6% in the intervention and control groups, respectively.

**Conclusion:**

The study showed that CRP testing in patients with acute cough/RTI may reduce antibiotic prescribing and referral for radiography, probably without compromising recovery.

**Trial registration:**

The trial was registered in the ClinicalTrials.gov Protocol Registration System (identification number: NCT01794819).

## Background

European medical professionals are concerned about the overuse of antibiotics and increased levels of bacterial resistance [1,2]. Lower respiratory tract infections (LRTI) and cough are two of the most common reasons in Europe for consulting a general practitioner (GP) [3]. Between 80% and 90% of all antibiotics are prescribed in primary care, mostly for respiratory tract infections (RTI) [1]. The frequency of antibiotic prescribing in patients with acute cough varies widely between European countries (from 28% to 92%) [4]. From a public health perspective, the major focus of the recent European Respiratory Society (ERS) guidelines for the management of LRTI is appropriate prescription of antibiotics [3].

In the Arkhangelsk region of the Russian Federation, community-acquired respiratory infections are common in general practice, constituting 40% of all consultations in adults [5]. About two-thirds of all antibiotics prescribed are for treatment of such infections. Selecting the right patients for antibiotic treatment is a major diagnostic challenge for general practitioners who are caring for patients with acute community-acquired LRTI, infectious exacerbations of asthma, or chronic obstructive pulmonary disease (COPD). The main problems in the management of patients with LRTI in primary care are, on the one hand, prescriptions of unnecessary antibiotics in cases of acute bronchitis where the infection is usually self-limiting, and, on the other hand, the risk of missing treatment of life-threatening pneumonia [6-8]. Differentiation between viral and bacterial LRTI would have therapeutic implications, but common clinical signs have low sensitivity and specificity for bacterial infection, and standard microbiological examinations are, in most cases, not practical in primary care [7]. Diagnostic uncertainty and an over-reliance on abnormal lung sounds on auscultation [3,9] can be reasons for overprescribing antibiotics in patients with acute cough, as are patient expectation and demand [10]. Evidence-based antibiotic prescribing can be promoted in several ways, one of which could be the application of point-of-care testing (POCT) for C-reactive protein (CRP), an acute-phase protein that shows increased levels in serum during infection and tissue damage [11].

Rapid tests for CRP were introduced into general practice about 20 years ago. They are widely used in Nordic countries, mostly in cases of upper respiratory tract infection (URTI) and LRTI (from 31% to 74% of cases) [8,9,12,13]. The antibiotic prescription rate is also relatively low in these countries, as shown in a European study carried out in a primary care setting [4,14]. Rapid CRP tests had not been used in primary care in the Arkhangelsk region before the start of this study.

In most cases, the CRP test cannot differentiate between bacterial and viral infections [15], but it does help to decrease diagnostic uncertainty [16]. Most patients consulting in general practice have CRP levels less than 20 mg/L [8,9]. By avoiding the administration of antibiotics to patients with such low CRP values, unnecessary use of antibiotics may be reduced [17]. Although the strong association between CRP value and the presence of pneumonia is well documented [6,7,18], evidence showing that the test can be used by GPs to improve rational use of antibiotics in LRTI is still sparse and uncertain [17,19-21]. However, the use of CRP testing in primary care has been recommended in the latest European guidelines for treatment of LRTI [3]. The Russian guidelines concerning community-acquired pneumonia recommend CRP testing as an optional investigation [22]. These guidelines indicate that chest radiography is mandatory when pneumonia is suspected [22].

A recently published systematic review and meta-analysis of primary care studies [23] pointed to a role of POCT for CRP in significantly reducing antibiotic prescribing at the index consultation for patients with RTIs.

The aim of this study was to evaluate the usefulness in Russian general practice of CRP testing in patients with acute cough/RTI. In addition to studying the effect of CRP testing on the prescription of antibiotics, we wanted to find out whether the frequency of referral for radiography could be reduced.

## Methods

### Setting

Eighteen general practitioners (GPs), nine from the Arkhangelsk region and nine from the Murmansk region, from both urban and rural offices, were randomized into intervention and control groups. The trial was conducted over 12 weeks (from 30 January to 30 April 2010). All registrations were made by GPs in their offices.

### Study population

Patients with acute cough/LRTI (including acute bronchitis, pneumonia, and infectious exacerbations of COPD or asthma) were included. Other inclusion criteria were age 18 years or older, an illness of less than 28 days duration, first consultation for the illness episode, being seen in a physician’s office, and written consent to participate. Exclusion criteria were an inability to fill out study documentation, being previously included in the study, immunocompromised status (HIV patients, immunosuppressive treatment), and ongoing treatment with oral corticosteroids.

### Desi**g**n

Cluster randomization was performed with GPs as units with SPSS 18.0 (IBM, Armonk, NY, USA). The rationale for using a cluster design was to ensure consecutive recruitment of patients and to avoid the situation where experience gained from the use of the test contaminated the care of control patients.

The second author generated the allocation sequence using SPSS, and the first author enrolled the clusters and made a list of clusters. Based on this list and using the allocation sequence, the first author assigned clusters to interventions.

All the GPs worked in separate outpatient departments (polyclinics), some in single GP offices and others within a GP partnership (with doctors who did not participate in this study). The sample sizes were based on a hypothesis of 20% reduction in antibiotic prescribing in the intervention group compared with the control group. Based on the chi-square statistic, the required sample size in each group was 72 participants (with a power of 90% and a risk of false positive difference less than 5%).

Two months before the trial, a baseline study without CRP testing was conducted that included 13 of the 18 participating GPs, using the same case report form (CRF) and examination. This allowed observation of prescription rates before and after the clinical trial, serving as a sensitivity analysis.

The CONSORT checklist and flow diagram are included as additional files.

### The case report form (CRF)

The CRF was similar to that used in the GRACE study (Genomics to combat Resistance against Antibiotics in Community-acquired lower respiratory tract infections in Europe [24]), describing symptoms, findings, and treatment in LRTI [4]. The GPs reported the following 15 symptoms in a questionnaire: cough, sputum production, shortness of breath, wheeze, coryza (blocked/runny nose), fever during this illness, chest pain, muscle aches, headache, disturbed sleep, feeling generally unwell, interference with normal activities, confusion/disorientation, and diarrhoea. If increased sputum production was reported, the colour of the sputum was recorded. Symptom severity scores were calculated using the scores for 13 symptoms (similar to that computed in the GRACE study) [4]. The categories for clinicians to rate the severity of each symptom as “no problem”, “mild problem”, “moderate problem”, or “severe problem” were scored 1, 2, 3, and 4, respectively. Scores were calculated for patients with a minimum of 85% (that is, 12 of 14 symptoms) of their symptoms recorded. This score was scaled to range between 0 and 100 so that it could be interpreted as a percentage of maximum symptom severity.

Other variables registered included sex, age and smoking status (never smoker, previous smoker and current smoker). For previous and current smokers, the average number of cigarettes per day and the number of smoking years were recorded. The pack-year criterion was calculated: one pack-year of smoking would mean that someone had smoked one pack of cigarettes (20 cigarettes) daily for one year.

The clinical examination included a chest examination and axillary temperature. The following chest findings were recorded: diminished vesicular breathing, wheezes, crackles and rhonchi.

After the clinical examination, the GPs recorded their provisional diagnosis, choosing from the following: URTI, acute bronchitis, pneumonia, COPD, asthma, non-infectious cough, and other diagnosis.

The comorbidities registered were: 1) pulmonary diseases, including COPD, asthma, tuberculosis, bronchiectasis, lung cancer, and other lung disease; 2) heart diseases, including heart failure, ischaemic heart disease, and other heart diseases (e.g., valvular lesions, cardiomyopathy); 3) diabetes; and 4) other chronic diseases.

### Optional examinations

Chest radiography was accessible for all patients, and other investigations (e.g., culture of sputum, spirometry, electrocardiogram) could be ordered when necessary. All suspected pneumonia cases were confirmed by chest radiography, and films were routinely reviewed by specialists at radiology departments.

### CRP testing

Before the clinical trial began, all GPs participated in two vocational training sessions concerning the CRP test, including theoretical and practical information. They were given guidelines about the interpretation of CRP results. This information included a summary of the literature on RTI and the role of CRP; paper cases of patients with different RTIs and different CRP values were discussed. They were told that antibiotics were usually not needed when the CRP value was below 20 mg/L and that a prescription could be indicated for CRP values above 50 mg/L, taking into account the duration of illness [15]. However, the management, including antibiotic treatment, should be decided for each patient on an individual basis.

The CRP test was performed in the intervention group at both the first and second consultations. The Afinion test system (Axis Shield) was used, which provides results within 5 minutes and before treatment is determined. This test is based on solid-phase sandwich immunometric analysis. The measurement range in whole blood samples is 8–200 mg/L. Test kits were supplied by Axis Shield.

### Treatment

GPs could prescribe any treatment, including antibiotics and other drugs for the cough (e.g., cough mixture) and additional medication if deemed necessary. They were told that medication should be prescribed after the clinical examination (and after the CRP test in the intervention group), without waiting for chest radiography results.

### Outcome measures

The primary outcome was the antibiotic prescribing rate. Secondary outcomes were referral to radiography and rate of recovery at the follow-up consultation after 2 weeks with the following five alternatives: “fully recovered”, “almost recovered”, “slightly improved”, “unchanged”, and “worse”. Reconsultations (another consultation with the GP within 2 weeks) and complications (in need of hospitalization) were recorded.

### Statistical analysis

CRP values were divided into three groups: CRP < 20 mg/L, 20–50 mg/L and ≥ 50 mg/L. Differences in the rate of prescribing antibiotics and referral to chest radiography were calculated, as was the percentage of participants who stated that they had recovered or almost recovered after 2 weeks. Differences in patient characteristics at inclusion between the intervention and the control group were analysed to check for recruitment bias. Changes in prescription rate between baseline and the clinical trial for each GP were analysed as part of the sensitivity analyses. The chi-square test was used to assess differences, and a P-value < 0.05 was considered to indicate significance. As a second sensitivity analysis, the predictive value of CRP testing for antibiotic prescribing was evaluated by multivariable logistic regression, in a model including other relevant explanatory variables.

Statistical analysis was performed using SPSS 18.0.

### Ethics

All patients gave informed consent. The study was approved by the local Ethics Committee of the Northern State Medical University (Arkhangelsk, Russia).

## Results

### Study population

The intervention group that underwent the CRP test consisted of 101 patients recruited from four GP offices in Arkhangelsk and four GP offices in Murmansk (51 and 50 patients, respectively). Initially, 98 patients were recruited to the control group. During analysis, it became clear that there were incomplete registrations in the CRFs from two GPs, and follow-up data were frequently missing, as were patient consent forms. To assure quality, all patients from these two GPs were excluded from the analysis, and we ended up with 78 patients in the control group from four GP offices in Arkhangelsk and five GP offices in Murmansk (51 and 27 patients, respectively). The mean age was 50.8 years in both groups. Feeling unwell and experiencing limitations in daily activities were recorded more frequently in the intervention group than in the control group, whereas the severity scores were similar in the two groups. The GPs in the control group reported more frequently than those in the intervention group that the patients wanted antibiotics (Table 1). The most frequent diagnoses were URTI (50% and 41% in the intervention and control group, respectively) followed by acute bronchitis. Pneumonia was confirmed in 7% of the patients in the intervention group and in 17% in the control group.

**Table 1:** **Patient characteristics and findings in primary care patients with acute cough categorized by CRP**^**1 **^**testing**

	**Intervention group, CRP tested (n = 101)**		**Control group, CRP not tested (n = 78)**		**P**
	**n**	**(%)**	**n**	**(%)**	
Male	29	(28)	20	(26)	0.7
Current smokers	28	(27)	16	(21)	0.3
Pre-existing illness:					
Pulmonary diseases	15	(15)	14	(18)	0.7
Heart diseases	17	(17)	3	(4)	0.007
Diabetes	5	(5)	3	(4)	1.0
Any comorbidity	55	(54)	39	(50)	0.6
Symptoms^2^:					
Cough	60	(59)	48	(62)	0.9
Sputum	20	(20)	21	(27)	0.3
Discoloured sputum	56	(57)	49	(63)	0.4
Shortness of breath	9	(9)	11	(14)	0.3
Wheeze (reported)	9	(9)	17	(22)	0.02
Coryza	19	(19)	9	(12)	0.2
Fever (history of)	31	(31)	26	(33)	0.7
Chest pain	5	(5)	5	(6)	0.7
Muscle aches	13	(13)	2	(3)	0.01
Headache	19	(19)	4	(5)	0.07
Disturbed sleep	10	(10)	4	(5)	0.3
Feeling unwell	47	(46)	15	(19)	0.0001
Interference with daily activities	44	(43)	15	(19)	0.0007
Symptom severity^3^	44		43		0.4
	(27–63)		(29–69)		
Findings:					
Diminished breath sounds	29	(29)	30	(38)	0.2
Wheezes	21	(21)	19	(24)	0.6
Crackles	3	(3)	2	(3)	1.0
Rhonchi	7	(7)	3	(4)	0.5
Any abnormal lung sound	60	(59)	54	(69)	0.2
Temperature ≥ 37.2°C	51	(50)	49	(63)	0.1
CRP ≥ 20 and < 50 mg/L	13	(13)			
CRP ≥ 50 mg/L	7	(7)			
Perceived patient preference for antibiotics^3^	10	(10)	18	(23)	0.02

^1^CRP = C-reactive protein.

^2^Reported by patient to be a moderate or severe problem.

^3^Symptom severity scores calculated using the scores for 13 symptoms that were summed, and median, minimum and maximum values (in brackets) calculated.

^4^The general practitioner agreed that the patient wanted her/him to prescribe antibiotics.

### Antibiotic prescribing

The rate of antibiotic prescribing on the day of inclusion was lower in the intervention group (37.6%) than in the control group (58.9%) (P = 0.006). The overall antibiotic prescribing rate during the two weeks was 40.6% in the intervention group and 71.8% in the control group (P = 0.0001).

**Figure 1:**
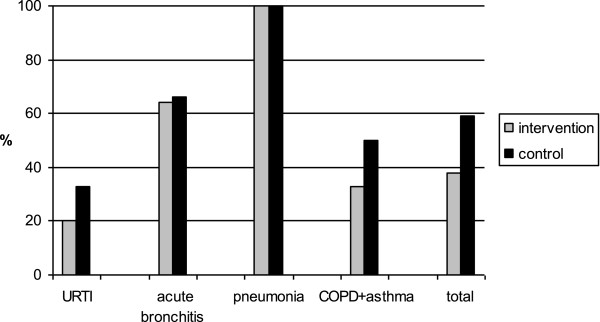
**Antibiotic prescribing by confirmed (X-ray) diagnosis in the intervention and control groups.** URTI = upper respiratory tract infections, COPD = chronic obstructive pulmonary disease.

Antibiotics were prescribed in all cases of suspected pneumonia (Figure 1). The second most frequent reason for prescribing antibiotics was acute bronchitis (64% and 68% in the intervention and control groups, respectively). The most frequently used antibiotic was amoxicillin, often in combination with clavulanic acid, prescribed for 73% and 72% of those given antibiotics in the intervention and control groups, respectively.

**Figure 2:**
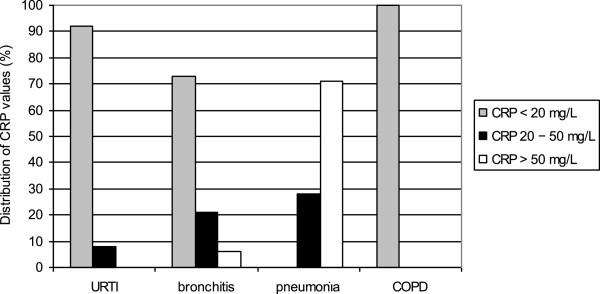
**Distribution (%) of CRP values by diagnosis in the intervention group.** CRP = C-reactive protein.

**Figure 3:**
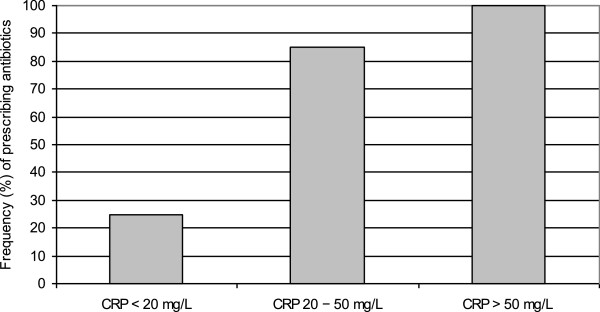
**Frequency (%) of prescribing antibiotics by CRP value in the intervention group.** CRP = C-reactive protein.

The mean CRP level in the intervention group was 11.5 ± 24.4 mg/L. Most of the patients with pneumonia had CRP values > 50 mg/L, whereas none of the URTI patients had such high CRP values (Figure 2). When the CRP value was higher than 20 mg/L, 85% of the patients were prescribed antibiotics, compared with 28% when the CRP value was below 20 mg/L (P = 0.0002) (Figure 3).

### Radiography

The referral rate for chest radiography was significantly lower in the intervention group (55.4%) than in the control group (76%) (P = 0.004). All patients with a clinical diagnosis of pneumonia were referred for radiography, whereas the lowest frequency of referral was for patients diagnosed with URTI (45% and 37% in the intervention and control groups, respectively).

### Recovery

The frequency of reporting “almost recovered” or “fully recovered” after 2 weeks was 91.1% in the intervention group and 92.3% in the control group, but “fully recovered” was most frequently reported in the intervention group (Table 2). All patients had recovered to some degree; no patients had become worse.

**Table 2:** Clinical recovery rate in the clinical trial

	**Intervention group, n (%)**	**Control group, n (%)**
Fully recovered	55 (54.4%)	26 (33.3%)
Almost recovered	37 (36.6%)	46 (58.9%)
Slightly improved	7 (6.9%)	5 (6.4%)

### Sensitivity analyses

#### Comparisons with the baseline study

Thirteen of the 18 GPs who took part in the clinical trial also participated in the baseline study I, which included 52 patients above the age of 18 in Arkhangelsk and 46 in Murmansk. The rate of referral for X-ray examination in the baseline study was 70.1%, and the antibiotic prescribing rate was 63.3%. The clinical recovery rate (the percentage of patients who were “fully recovered” or “almost recovered”) was 87.7%. Antibiotic prescribing rates in the baseline study and in the clinical trial by the four GPs randomized to the control group and the seven GPs randomized to the intervention group are compared in Table 3. A reduction in the prescribing rate of five of seven GPs in the intervention group was observed, whereas only one of the six GPs in the control group had reduced their prescribing rate.

**Table 3:** Antibiotics prescribed in the baseline study and the clinical trial by GPs participating in both

	**Baseline**			**Clinical trial**			
	**Number of patients**	**Patients treated with antibiotics**	**Antibiotic prescribing rate (%)**	**Number of patients**	**Patients treated with antibiotics**	**Antibiotic prescribing rate (%)**	**P**
Control group							
GP A	6	2	(33)	12	8	(66)	0.3
GP B	4	2	(50)	14	7	(50)	1.0
GP C	6	4	(66)	8	8	(100)	0.2
GP D	5	5	(100)	8	8	(100)	1.0
GP E	6	2	(33)	10	6	(60)	0.6
GP F	7	6	(86)	10	7	(70)	0.6
Total	34	21	(62)	62	44	(71)	0.4
Intervention group							
GP G	7	2	(28)	19	8	(42)	0.7
GP H	6	5	(83)	10	4	(40)	0.1
GP J	5	5	(100)	10	6	(60)	0.2
GP K	7	2	(28)	12	1	(8)	0.5
GP L	9	3	(33)	10	0	(0)	0.1
GP M	7	7	(100)	10	4	(40)	0.08
GP N	6	4	(66)	10	7	(70)	1.0
Total	47	28	(59)	81	30	(37)	0.02

#### Evaluating the intervention by multivariable analysis

When the effect of the intervention was analysed by multivariable logistic regression with antibiotic prescribing as outcome variable and allocation to CRP testing, URTI diagnosis, symptom score, any chest finding, any comorbidity, and perceived patient preference for antibiotic as explanatory variables, the OR of allocation to CRP testing was 0.4 (95% confidence interval [CI] 0.18–0.89). Symptom score and perceived patient preference for antibiotics were significant positive predictors of antibiotic prescribing in this model, whereas URTI diagnosis was a significant negative predictor.

## Discussion

This study demonstrated that the rate of antibiotic prescribing and referral to radiography could be reduced by the introduction of POCT for CRP. This reduction was most likely obtained without decreasing the patient recovery rate.

Some previous studies have failed to find an effect of CRP testing on antibiotic prescribing [19,20]. In a study from 1995 by Melbye and co-workers, the use of a rapid CRP test did not lead to a reduced rate of antibiotic prescribing in patients with LRTI [20]. Low levels of trust in the test at that time probably led to frequent prescribing, even in patients with low CRP values. A study by Gonzales and co-workers also indicated that the CRP test provided no additional value beyond clinical decision support in terms of reducing antibiotic use in adults with acute cough [19]. In this study, patients with a CRP level as low as 10–20 mg/L could be treated with antibiotics according to the algorithm in the CRP group, which may be one reason for the increased prescribing rate in those tested for CRP.

In contrast to the negative findings of these two studies, Cals and co-workers showed that the use of CRP testing significantly reduced antibiotic prescribing for LRTI without decreasing the quality of care and the outcome of treatment [25]. The results of our study were similar to those of Cals et al., who also found that multifaceted interventions in addition to the use of the CRP test gave additional benefits: they found that it was valuable to provide guidance to GPs in communication skills [25]. In a recent study of 621 adult patients with acute cough or fever, 20.5% of whom had radiographically confirmed pneumonia, Steurer and co-workers concluded that pneumonia could safely be excluded in patients with CRP values below 10 mg/L and in patients without dyspnoea or daily fever with values between 11 and 50 mg/L [21]. The CRP test has also recently been found to be useful in identifying patients with COPD exacerbations who do not need antibiotic treatment [26].

In 2012, Engel et al. published a systematic review titled “Evaluating the evidence for the implementation of C-reactive protein measurement in adult patients with suspected lower respiratory tract infection in primary care” [27]. Most of the studies that were reviewed showed limited evidence for the usefulness of CRP measurement in adult patients in primary care with suspected LRTI [27]. Only one study (Cals et al. [25]) provided firm evidence that a reduction in antibiotic prescriptions could be achieved when CRP measurement was applied [27].

The CRP test may be most useful in patients with an intermediate risk of pneumonia. Van Vugt et al., when evaluating the CRP test, concluded that “A clinical rule based on symptoms and signs to predict pneumonia in patients presenting to primary care with acute cough performed best in patients with mild or severe clinical presentation” [28]. The prevalence of pneumonia in our study was somewhat higher than in the study by van Vugt et al. This can possibly be explained by seasonal variation in morbidity (winter–spring). The geographical location of the participating GPs (northern part of Russia) and the absence of vaccination against pneumococcal infections in this area can probably also be taken into account.

In a recent meta-analysis of 13 studies in primary care including 10,005 patients, CRP testing led to significantly reduced antibiotic prescribing at the index consultation. However neither the reduction in antibiotic prescribing at any time during the 28-day follow-up period nor the increase in patient satisfaction was significant [23]. The authors emphasized that future studies are needed to analyse the confounders that lead to this heterogeneity.

### Strengths and weaknesses of the study

In the Arkhangelsk region, POC testing for CRP had not been used in primary care before this study. GPs may need time to become more experienced in the use of CRP testing and to become confident in interpreting the results. The documentation of the test’s diagnostic properties and its popularity in primary care in Nordic countries may have made it easier for the GPs in the intervention group to rely on the test results.

The cluster randomization with a small number of patients in each unit made it more likely that differences between the intervention group and the control group could occur [29]. However, the uncertainty was reduced by finding a similar effect of the intervention in multivariable analysis. The results were also supported by the comparison with the baseline study. The fact that the GPs did not use CRP testing in their practice before the study made this comparison possible.

Recovery rates were evaluated by the GP and the patient together at the follow-up consultation after two weeks. GPs were aware of the purpose of the trial, and they recruited and treated patients and assessed the clinical outcome together with patients in an unblinded fashion and without standardized criteria. Because of this, the recovery data have to be interpreted with caution. Regrettably, we were missing data on the duration of illness at randomization.

### Clinical implications

CRP results have been demonstrated to influence strongly the decision about whether to prescribe antibiotics for acute cough, with a steep increase in prescribing with increased CRP values [8,9,12]. However, when GPs think that the patient wants antibiotic treatment, they frequently prescribe antibiotics in spite of a low CRP value [9]. Communication skills are important in order to convince the patient that antibiotics are not needed. Results from CRP testing may be helpful in such an argument [9]. Using CRP testing in addition to a thorough physical examination can develop more trust between patient and doctor, and improve satisfaction with the consultation [17].

The new European guidelines for the management of adult LRTI do not indicate strong scepticism about the use of the test [3]. According to these guidelines, CRP testing can be done in patients with suspected pneumonia [3]. In cases of persistent doubt after CRP testing, a chest X-ray should be considered to confirm or reject the diagnosis [3].

## Conclusions

Our study confirms that the use of POCT for CRP may reduce the rate of antibiotic prescription for acute cough/RTI. Careful use and interpretation of CRP testing in patients with RTI has the potential to benefit patients and to help GPs in the important struggle against antibiotic resistance.

## Competing interests

The authors declare that they have no competing interests.

## Authors’ contributions

EA participated in the design of the study, led the data collection, performed the statistical analysis and drafted the manuscript. HM conceived the study and participated in its design and coordination, and helped to draft the manuscript. Both authors have read and approved the final manuscript.

## Pre-publication history

The pre-publication history for this paper can be accessed here:

http://www.biomedcentral.com/1471-2296/15/80/prepub
